# Interactive and evolutionary effect of CASZ1 gene variants on varicose veins susceptibility in South Asian Indians

**DOI:** 10.1186/s40659-025-00599-1

**Published:** 2025-03-19

**Authors:** Rohit Mehra, Vikram Patra, Rishi Dhillan, Dattatraya CVNM, Hemender Singh, Love Gupta, Garima Rastogi, Indu Sharma, Varun Sharma

**Affiliations:** 1 Department of Vascular and Endovascular Surgery, Command Hospital (Southern Command), Pune, India; 2 Department of Vascular and Endovascular Surgery, 92-Base Hospital, Jammu, Kashmir India; 3 Department of Vascular and Endovascular Surgery, Army Hospital (Research and Referral), New Delhi, India; 4 NMC Genetics India Pvt. Ltd, Gurugram, Haryana India; 5 Keck School of Medicine, University of Southern California, Los Angeles, USA; 6 Head Research and Development Division, NMC Genetics India Pvt. Ltd. Gurugram, Gurugram, India

**Keywords:** Varicose veins, CASZ1 gene, Chronic venous diseases, South Asian Indians

## Abstract

**Background:**

Varicose veins (VV) are spectrum of common vascular diseases having complex genetic etiology. The Castor Zinc Finger 1 (*CASZ1*) gene has been involved in vascular development and its variant has shown association with VV in various ethnicities, but *CASZ1* susceptibility to VV risk is unexplored in the South Asian Indian population. The objective of this study was to estimate the association of the CASZ1 gene variations and VV susceptibility in the South Asian Indians, and to examine the evolutionary patterns of these variants compared to other populations.

**Methodology:**

Population based case control analysis was conducted on all *CASZ1* variants present in the Global Screening Array, including the established VV variant rs11121615 with a focus on validating and identification of both novel and established genetic markers to capture a full spectrum of population-specific genetic markers unique to studied population group. Linkage disequilibrium patterns and cumulative variant effects were also analyzed, followed by selection pressure assessment using neutrality tests.

**Results:**

Three *CASZ1* variants rs72860191 (OR 1.58, 95% CI 1.07–2.32, *p* = 0.01), rs7519604 (OR 1.43, 95% CI 1.05–1.94, *p* = 0.01), and rs11121615 (OR 0.69, 95% CI 0.50–0.95, *p* = 0.02) were observed to be significantly associated with VV. Haplotype analysis identified unique haplotype structure of South Asian Indians compared to other global populations. Moreover, the cumulative OR was observed to be higher than the independently estimated values (OR = 2.41, 95% CI 1.48–3.94), indicating genotypic epistasis of VV associated variants. The neutrality tests revealed balancing selection within *CASZ1* in the studied population compared to other populations,

**Conclusion:**

The present study identified *CASZ1* variants and their epistatic interactions is associated with VV susceptibility supported with evidence of balancing selection, provides crucial insights into the genetic architecture of VV in studied group, highlighting the impact of evolutionary forces on disease susceptibility.

**Supplementary Information:**

The online version contains supplementary material available at 10.1186/s40659-025-00599-1.

## Introduction

Varicose Veins (VV) is a part of the spectrum of chronic venous disorders (CVD) which have plagued mankind since times immemorial. Though a ubiquitous disease it has a unique expression in humans. Despite quantum leaps in the understanding of VV the etiological factors remain illusory [1, 2]. The risk factors implicated in development of VV include gender, age, family history, lifestyle, prolonged standing, hereditary risk factors, pregnancy and deep venous thrombosis [3]. This ubiquitous entity has a reported prevalence of 10–20%, in the western literature, while the corresponding Indian prevalence has been reported as 5% [4, 5].

Castor zinc finger 1 (*CASZ1)* plays a role in vascular assembly and lumen morphogenesis [6, 7], which is crucial for normal vein structure and function. Its regulation effects the physiological development of veins [8], potentially leading to condition like VV and other venous disorders. Heredity has been reported to play an important role in the pathogenesis of VV despite the genetic basis not been completely elucidated. In the recent years, few genomes wide studies have tried to broaden the understanding providing valuable insight in the role of common genetic variants in VV pathophysiology [9–11]. Amongst the role of common genetic variant, CASZ1 gene variation has been observed to be highly significant (*p* < 5 e^− 08^) and validated in various other populations [10–12].

Despite the proven significance of *CASZ1* variants in VV susceptibility across various ethnicities, there is scanty genetic information of VV available in literature for South Asian Indians. The present study was formulated with the objective of examining *CASZ1* association with VV. Furthermore, the study examined evolutionary patterns of *CASZ1* variants through comparative population analysis. The present study provides crucial insights into population-specific genetic risk factors and the evolutionary forces shaping VV susceptibility in South Asian Indians.

## Materials and methods

### Sample collection and criteria

A total of 548 individuals (103 VV cases and 445 controls) were included in the study. Most of participants were from North India and had resided in their respective regions for at least three generations. Consenting patients attending the out-patient department of a tertiary care hospital were recruited in the study. These patients were enrolled after a thorough lower limb clinical assessment for chronic venous disease (CVD), and were classified using the Clinical, Etiological, Anatomical and Pathological (CEAP) classification system that is based on clinical manifestation of CVD [13]. All the patients were examined by experienced vascular surgeons and individuals with trait or family history of VV were excluded from the cohort. For the consenting patients’ blood samples (2 ml) was collected by a phlebotomist in a bar-coded EDTA vacutainer, under full aseptic conditions and the vacutainer stored at 4 °C. The individual patient record was compiled simultaneously. The study was approved by institutional ethical committee.

### DNA isolation and genotyping

DNA extraction from the blood samples was performed using the QIAGEN DNA Mini kit and their quality and concentration was checked on 1% Agarose gel. Samples which passed the quality check were further processed on the protocol of Illumina high-throughput screening assay (HTS) for the genotyping. Global screening array bead chip was used to generate genotype data. The observed call rate for all individuals included in the analysis exceeded 98%. The data so obtained was analyzed using plink 1.09 [14]. To validate the genetic affinity of the studied cohort with global populations, a principal component analysis (PCA) was performed using *–pca* command. For evaluating the CASZ1 gene in the bead array chip, a total of seventy-two variations were extracted using *- extract* command and were filtered (*--mind 0.05 --maf 0.05 --geno 0.1*). Additionally, Hardy Weinberg Equilibrium (HWE) was examined and variants avoiding HWE and frequencies filter were excluded from the study. In total, fifty-six variations passed the quality check (QC). Further, the QC passed variants were evaluated for association test using *--assoc --ci 0.95* after correction with ethnicity, age and gender. The linkage disequilibrium (LD) was estimated using Broad institute’s Haploview tool [15]. Locus in strong LD and showing association (p value < 0.05) were examined for cumulative risk using model stated elsewhere [16]. To assess the evolutionary influence of *CASZ1*, neutrality test was conducted with the DNAsp multi-MSA plugin, enabling comparison of selective pressures between the studied population and global populations within the standard neutral model framework [17, 18]. The power of the study was calculated using Genetic Association Study (GAS) calculator [19].

## Results

A total of 548 patients were evaluated in the study. The clinical characteristics and demographic profile of the cohort is summarized in Table 1. The significant differences were observed between cases and controls. Cases were younger (47.5 ± 17 vs. 52.3 ± 15 years, *p* = 0.02) and moderately weightier (71.2 ± 14 vs. 68.1 ± 14 kg, *p* = 0.05), suggesting earlier onset and a potential link to weight (Table 1). No differences were observed in height (*p* = 0.63). A smaller proportion of cases reported a history of smoking (27.2% vs. 38.7%), and 59.2% had standing occupations, supporting the hypothesis of prolonged standing as a risk factor. Clinically, 11.6% of cases presented with deep vein thrombosis, while most were classified as CEAP stage C2 (53.4%), indicating moderate disease severity.

**Table 1:** Clinical and demographic characteristics of the studied cohort

Clinical Characteristics	Case (*N* = 103)	Controls (*N* = 445)	*p* value
Age (in years) (SD)	47.5 (17)	52.3 (15)	0.02*
Weight (in kilograms) (SD)	71.2 (14)	68.1 (14)	0.05
Height (in centimeters) (SD)	168.1 (8.9)	168.6 (10)	0.63
Male (in percentage)	67.9	65.4	--
Female (in percentage)	32.0	34.6	--
History of smoking (in percentage)	27.2	38.7	--
Standing occupation (in percentage)	59.2	--	--
Deep Vein Thrombosis (in percentage)	11.6	--	--
CEAP Classification (in percentage)			
C1	7.8	--	--
C2	53.4	--	--
C3	14.6	--	--
C4	10.7	--	--
C5	8.7	--	--
C6	4.9	--	--

*SD = Standard Deviation

The results from PCA validates that the genetic profiles of the study individuals cluster aligns within the South Asians (SAS) (Supplementary Fig. 1). The observed genetic homogeneity of the studied cohort emphasizes their significance as a representative sample of SAS for studying CASZ1 variants associated with VV. Posts filtering fifty-six variations passed data quality check and were proceed for association analysis. Three variants of CASZ1 gene (rs72860191, rs7519604 and rs11121615) were showing association with VV in studied population cohort. The odds ratio (OR) observed for variant rs72860191 was 1.58 (95% CI 1.07–2.32), p value 0.01. Other two variants that showed significant association were rs7519604 and rs11121615 and the OR of 1.43 and 0.69, respectively. The variant rs7519604 and rs72860191 were providing risk whereas the effect allele (minor allele) of variant rs11121615 showed protection against VV in studied population group (Table 2). All other variants information has been summarized in supplementary Table 1.

**Table 2:** Effect allele frequency distribution and *CASZ1* variant showing association with VV in Indian population

CHR	SNP	Variation Reported in dbSNP	Minor Allele reported in dbSnp (SAS)	EA (Allele Orientation)	EAF	*p* value*	HWE *p* value	OR (95% CI)
1	rs72860191	G > A,C	A	A (Reverse Strand)	0.20	0.01	0.2	1.58 (1.07–2.32)
1	rs7519604^$^	T > C	T	A (Reverse Strand)	0.56	0.01	0.2	1.43 (1.05–1.94)
1	rs11121615^$^	C > T	T	A (Reverse Strand)	0.34	0.02	0.4	0.69 (0.50–0.95)
1	rs205488^$^	T > C	T	A (Reverse Strand)	0.45	0.12	0.8	1.27 (0.93–1.72)

CHR- chromosome, SNP- Single nucleotide polymorphism, EA- Effect allele, EAF- Effect allele Frequency, SNP - Single nucleotide polymorphism, OR– Odds ratio, * - corrected with age, gender and ethnicity, ^$^ - SNPs in strong linkage disequilibrium of *CASZ1* (D` >0.97)

**Fig. 1:**
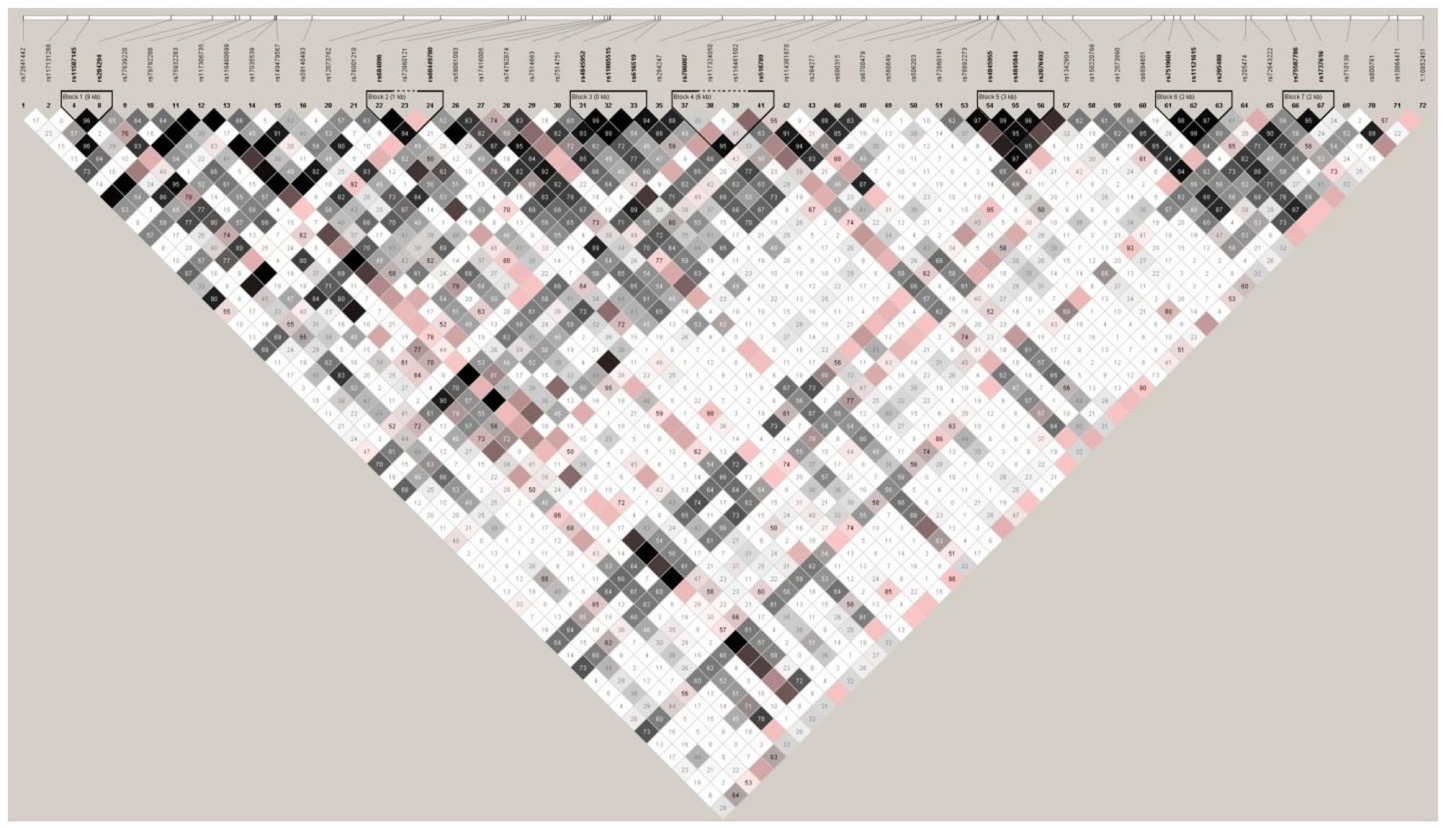
Haplotypes blocks observed after evaluating seventy-two variations of CASZ1 gene in South Asian Indians. The linkage disequilibrium values are presented in D`

The haplotype analysis was implemented to weigh haplotype pattern in studied cohort. Haplotype analyses identified seven haplotype blocks (Fig. 1). Interestingly sixth haplotype block had two variations that have shown association. These variations were rs7519604 and rs11121615. Third variation in this sixth block i.e., rs205488 has not shown association with VV (Table 2). The linkage disequilibrium (D`) observed for haplotype block was greater than 0.90. In order to evaluate the cumulative risk of significantly associated variants, interactive analyses was performed by providing weightage to risk alleles (Table 3).

**Table 3:** Haplotype analysis of *CASZ1* variants and their cumulative effects on VV susceptibility

Haplotype Genotypes	CES OR (95% CI)	*p* value
rs7519604_AA + rs11121615_GG + rs205488_AA	1.6 (1.03–2.68) ^**^**^	0.03
rs7519604_AA + rs11121615_GG + rs72860191_AA	2.41 (1.48–3.94)^#^	0.0003

^^^Model 1 = rs7519604_AA + AG + rs11121615_GG + AG + rs205488_AA + AG vs. all genotypes (SNPs in Strong LD)

^#^ Model 2 = rs7519604_AA + AG + rs11121615_GG + AG + rs72860191_AA + AG vs. all genotypes

CES- Cumulative effect of SNPs

Presence of two significant associated variations in sixth locus pinpoints the locus of interest in CASZ1 gene. To assess the cumulative risk of locus and taking the dominant models to measure the maximum effect of risk allele in population, two models were hypothesized: Model 1 = rs7519604_AA + AG + rs11121615_GG + AG + rs205488_AA + AG vs. all genotypes (sixth haplotype locus variants with LD > D` 97). The OR observed for model 1 was 1.6 (95% CI 1.03–2.68). However, for model 2: **=** rs7519604_AA + AG + rs11121615_GG + AG + rs72860191_AA + AG vs. all genotypes (two significantly associated variants from sixth locus and rs72860191 from another locus), the OR observed was 2.41 (95% CI1.48-3.94, p value 0.0003 ) (Table 3), surpassing the Bonferroni threshold (α = 0.05/56 = 8.93 × 10^− 4^). Statistical power was calculated under a multiplicative disease model, assuming prevalence of 15% [20] with an OR of 1.6 and risk allele frequency of 20%. With a significance threshold of 0.05, the study attained a power of 0.78 to detect true associations.

## Discussion

CASZ1 (OMIM: 609895) is required for direct regulation of EGFL7/RhoA mediated pathways that plays a key role in vascular system development in vertebrates and in cardiac as well as in vascular lumen differentiation [6]. The variation rs11121615 (1:10825577; GRCh37) in *CASZ1* has shown strongest association with VV in studies conducted on large cohorts [10, 12]. The same variant when evaluated in a Russian cohort and UK biobank expressed captivating results as the variant showed protection against VV in both the cohorts [21]. The level of significance observed was less (p value = 0.02) in the Russian cohort whereas in UK biobank the level of significance was surpassing GWAS threshold i.e., p value = 3.0e ^− 76^. Moreover, the variant was replicated in Netherland and New Zealand cohorts and the observed p value was 1.0e^− 2^ and 9.9e^− 6^, respectively [22]. Meta-analysis conducted on Russian, 23andme cohort, UK biobank, New Zealand and Netherland achieved highly significant p value of 1.5e^− 90^ [22]. Above stated genome wide as well as replication meta-analysis studies along with functional effect of CASZ1 provided sufficient indication that CASZ1 plays key role in VV pathophysiology. Interestingly, variant rs11121615, which is implicated as a risk causing genetic factor for VV in the world population, was showing protection in this studied Indian cohort. This findings are in concordance with clinical characteristics (Table 1), showing that most cases are younger than the control group. Moreover, the marginally higher weight in the cases compared with controls suggests that there may be a metabolic and possibly vascular component to risk predisposition, potentially and partially counterbalance by the risk-reducing effects of genetic variations. In addition, the high proportion of standing jobs among cases at 59.2% strengthens the multifactorial nature of VV where genetic variants of *CASZ1* may be interacting with occupational risks to define disease progression in the Indian cohort. This exclusive interaction of genetic predisposition and environmental impact might be one of the factors explaining the lower incidence of severe VV as noted in the Indian population compared with its Western counterparts [4, 5].

The results from the present study identified rs11121615 (G > A Reverse strand, C > T Forward Strand) which shows protective effect against VV with allelic OR 0.69 at 95% CI 0.50–0.95, p value 0.02. Minor allele “A” (reverse strand) was observed to be providing protection against VV. When considering strand orientation, our observations are fully consistent with these prior studies. Specifically, the protective A allele in our dataset corresponds to the T allele on the forward strand, while the risk G allele corresponds to the C allele on the forward strand (Table 1).

Interestingly, another variation rs7519604 (A > G), allele “A” that is in the strong LD with rs11121615 indicated risk of VV with observed OR 1.43 at 95% of 1.05–1.94, p value 0.01. In addition, rs72860191 (G > A) showed association with VV (OR 1.58 at 95% CI 1.07–2.38; p value 0.01). We have observed total three variants in CASZ1 gene showing association with VV in this Indian cohort.

We further estimated the combined effect of associated variants and two models were proposed. Model 1 used dominant model for variants *rs7519604_AA + AG + rs11121615_GG + AG + rs205488_AA + AG* (strong LD variants) and the observed OR was higher i.e., 1.6 at 95 CI 1.03–2.68. Model 2 comprised of dominant risk allele combinations of *rs7519604_AA + AG + rs11121615_GG + AG + rs72860191_AA + AG* (all significantly associated variants), the OR observed was 2.41 at 95 CI 1.48–3.94, p value 0.0003 (Table 3). The combined estimated OR was higher than independently estimated and Bonferroni-corrected threshold (α = 0.05/56 = 8.93 × 10^− 4^) which emphasized the importance of understanding the *‘genotypic epistasis’* of risk-attributing variations in a gene.

Although, variations residing in region Chromosome 1: 10,804,219, 10,824,835, 10,825,577 are conserved in primates (supplementary Fig. 2), yet the cumulative risk significance is less as compared with the other studies of VV in different cohorts [10, 12, 21, 22]. One of the plausible reasons could be the presence of a protective as well as risk causing variation in same haplotype which leads towards the balance selection or a differential risk allele frequency in South Asians, a plausible outcome of ancient admixture or haplotype differentiation in other populations. This could be an outcome of founder effect or distribution of population specific variants in South Asian Indians and other populations, resulting in distinct combined effects across populations *perse* [23, 24].

**Fig. 2:**
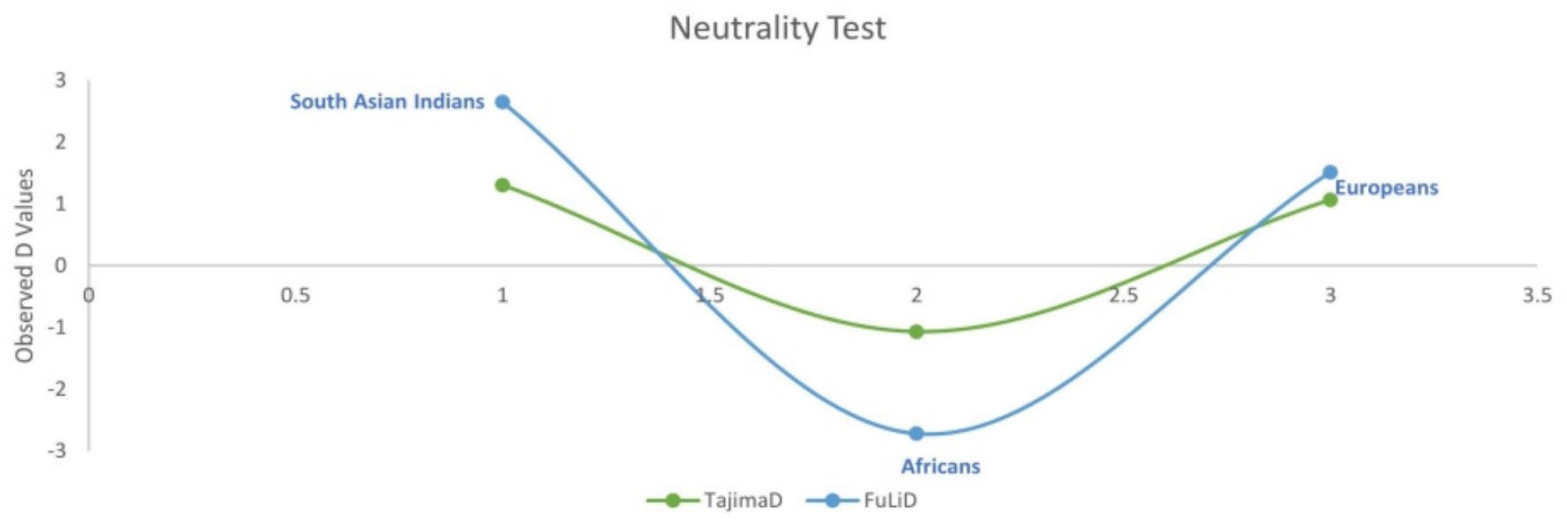
Tajima’s D and Fu and Li’ D test values are higher in Indian population than Europeans and Africans indicating balancing selection in CASZ1 studied region

To validate this assumption, we derived the variations from same location (Chr1: 1:10697392–10852451) of different populations (African and Europeans) from 1000 genome project (supplementary Fig. 3), the estimated haplotype structure of African and European populations was entirely different than the haplotype structure observed in South Asian Indians (supplementary Fig. 3). We speculate that the differences in haplotypic structure may also be the reason that the South Asian Indians have developed less severity towards the VV. Neutrality test was performed to understand the selection pattern using Tajima’s D and Fu and Li’s test in studied population set (South Asian Indians), African (as reference population) and in European population set from 1000 Genome data [25, 26]. D values observed for both the tests were higher in the studied population (Fig. 2) than Africans and Europeans indicating balancing selection in the studied region of CASZ1 gene.

This is the first comprehensive study on *CASZ1* variants in relation to VV susceptibility and detecting unique genetic and evolutionary dynamics. The interaction of the established rs11121615 along with two other novel loci (rs72860191, rs7519604) with significant cumulative effects, underscores that the combined effect of these common variants impacts more substantially to VV susceptibility. This presence of diverse gene variants within the *CASZ1* indicates evolutionary preservation due to balancing selection [27]. This genetic landscape showed potential heterozygote advantage to confer protection from the severe forms of any complex trait aiding in higher fitness [28, 29]; as evident by the predominance of milder CEAP classifications among studied cases (Table 1). The present study is focused on the South Asian Indian, which may bind the results to other ethnic groups. Further validation on other ethnic cohort warranted to ratify the observed associations and evolutionary insights. However, the haplotype differentiation provide evidence that the *CASZ1* variant distribution has been shaped by evolutionary. These findings highlight the importance of population-specific genetic risk profiling for VV and open up new opportunities for understanding genetic mechanisms and evolutionary context of vascular disease.

## Electronic supplementary material

Below is the link to the electronic supplementary material.

Supplementary Material 1

## Data Availability

Data supporting the findings of this study are available within the manuscript and its supplementary information files, with additional raw data accessible from the corresponding author upon reasonable request.
