# Supplementary material for: Interactive and evolutionary effect of CASZ1 gene variants on varicose veins susceptibility in South Asian Indians
Source: Biol Res. 2025 Mar 19;58:17. doi: 10.1186/s40659-025-00599-1 (PMC11921479; doi:10.1186/s40659-025-00599-1)
Supplement: Supplementary file 1 — Supplementary Material 1 [file 40659_2025_599_MOESM1_ESM.pdf]

CHR	SNP	Location	MA	MAF	P	OR	L95	U95
1	rs72641442	10697392	A	0.03883	0.2899	0.6647	0.3105	1.423
1	rs117131266	10702266	A	0.03883	0.4796	0.7587	0.3521	1.635
1	rs11587145	10704688	A	0.2039	0.8708	0.9693	0.6659	1.411
1	rs4845941	10705110	A	0.02913	0.927	0.9589	0.3907	2.353
1	rs284294	10713765	A	0.432	0.8713	1.026	0.7552	1.393
1	rs77639220	10718377	A	0.03883	0.3447	0.6935	0.3233	1.488
1	rs79792288	10720905	A	0.004854	0.3509	0.3898	0.05004	3.036
1	rs75932283	10721402	A	0.02427	0.1153	0.4783	0.1873	1.222
1	rs17035539	10725387	G	0.1019	0.7016	1.104	0.6663	1.828
1	rs149479567	10725469	A	0.004854	0.1471	0.2505	0.03315	1.893
1	rs58140483	10729381	A	0.09223	0.8852	0.9623	0.5708	1.622
1	rs113222733	10738871	A	0.02913	0.7318	0.856	0.3515	2.084
1	rs12073762	10739523	A	0.02913	0.2795	0.6212	0.2601	1.483
1	rs76001210	10743666	A	0.004854	0.4745	0.4775	0.06016	3.79
1	rs684096	10751153	A	0.1165	0.9021	1.03	0.6417	1.654
1	rs72860121	10752579	C	0.004854	0.4076	0.4293	0.05464	3.372
1	rs60449700	10752990	G	0.09709	0.8255	1.06	0.6336	1.772
1	rs2387232	10759741	A	0.04854	0.8536	0.9361	0.4643	1.888
1	rs59061093	10760472	A	0.1068	0.7332	1.09	0.665	1.786
1	rs17416005	10761759	A	0.2427	0.7888	0.953	0.6697	1.356
1	rs7514663	10762349	A	0.09709	0.9446	1.018	0.61	1.7
1	rs7514751	10762432	A	0.301	0.704	1.066	0.7657	1.485
1	rs4845952	10767379	A	0.3398	0.6102	1.087	0.7887	1.498
1	rs11805515	10767785	G	0.1893	0.8665	1.034	0.7016	1.523
1	rs616519	10768002	A	0.4854	0.6828	1.065	0.7866	1.443
1	rs284247	10774856	G	0.3301	0.6216	1.085	0.7852	1.499
1	rs706007	10780727	G	0.2816	0.8367	0.9652	0.6895	1.351
1	rs117324050	10783914	C	0.01456	0.661	0.7589	0.2203	2.614
1	rs140388766	10785145	A	0.06311	0.3849	1.327	0.6998	2.516
1	rs518789	10787538	A	0.432	0.6299	0.9276	0.6833	1.259
1	rs114361870	10787940	A	0.009709	0.9536	0.9553	0.2049	4.455
1	rs284277	10790797	C	0.4223	0.5562	1.097	0.8065	1.491
1	rs6540946	10793411	A	0.004854	0.2613	0.3291	0.04281	2.53
1	rs137924953	10794914	A	0.004854	0.226	0.3052	0.03991	2.334
1	rs880315	10796866	G	0.3883	0.3228	1.17	0.8566	1.599
1	rs138444091	10798295	A	0.01456	0.1023	0.3838	0.1166	1.264

1	rs6700479	10800737	A	0.2573	0.4741	1.136	0.8013	1.61
1	rs560649	10803357	G	0.2108	0.1208	1.35	0.9231	1.974
1	rs506203	10803450	C	0.2184	0.9837	0.9962	0.6904	1.437
1	rs76892273	10805263	G	0.06796	0.931	0.9738	0.534	1.776
1	rs4845955	10805414	G	0.2816	0.4756	1.131	0.8061	1.587
1	rs4845844	10805465	G	0.165	0.4913	1.156	0.7652	1.745
1	rs2076492	10808587	G	0.2184	0.3131	1.21	0.8351	1.753
1	rs1342904	10810317	A	0.06796	0.6781	0.8814	0.4855	1.6
1	rs150220766	10813714	A	0.004854	0.5535	0.5378	0.0668 9	4.324
1	rs12073890	10822357	A	0.08738	0.6485	0.8837	0.5192	1.504
1	rs205474	10832861	G	0.4709	0.0585 4	1.341	0.9889	1.819
1	rs75587786	10837378	A	0.09709	0.8621	0.9558	0.5739	1.592
1	rs1737616	10840054	A	0.09223	0.5094	0.8404	0.501	1.41
1	rs710138	10844444	A	0.2816	0.8473	0.9675	0.691	1.355
1	rs800761	10848699	A	0.1796	0.0808 9	1.432	0.9553	2.148
1	rs10864471	10849645	G	0.1117	0.2401	1.343	0.82	2.199

Supplementary Table 1: *CASZ1* variants evaluated for Varicose Veins in South Asian Indians. All these variations have not shown susceptibility towards the Varicose Veins in the studied population group, New Delhi, India, 2022-2024, South Asian Indians.

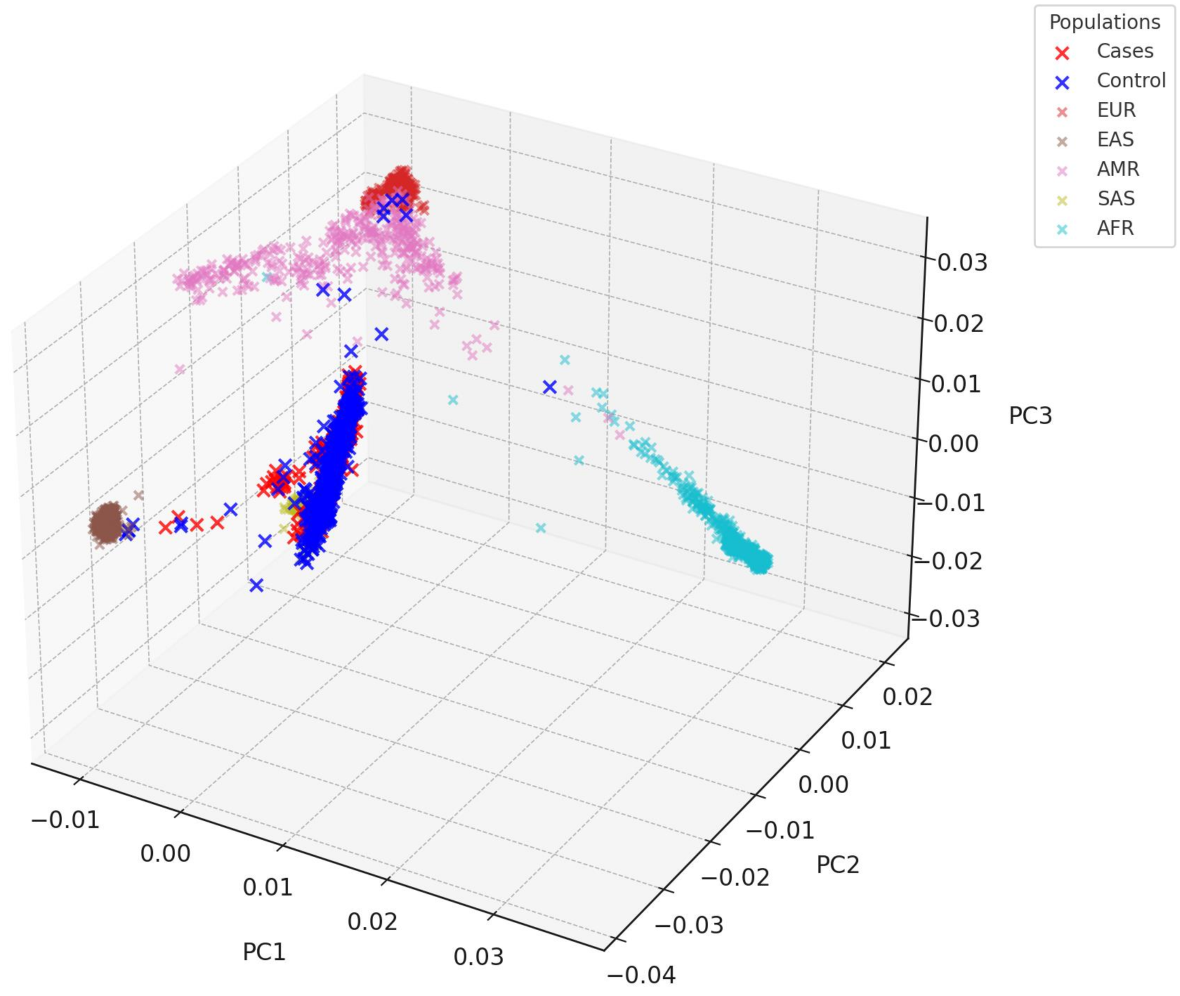

Supplementary figure 1: The PCA plot shows that the genetic profiles of the studied cohort (highlighted cases (red) and controls (blue)) aligned within the South Asian (SAS) population group. This alliance confirms the genetic homogeneity of the participants with the South Asian ancestry group, distinguishing them from other global populations such as Europeans (EUR), East Asians (EAS), Africans (AFR), and Americans (AMR). The clustering supports the population-specific relevance of the genetic analysis conducted in the study, ensuring that the observed associations for *CASZ1* variants are representative of the South Asian genetic landscape.

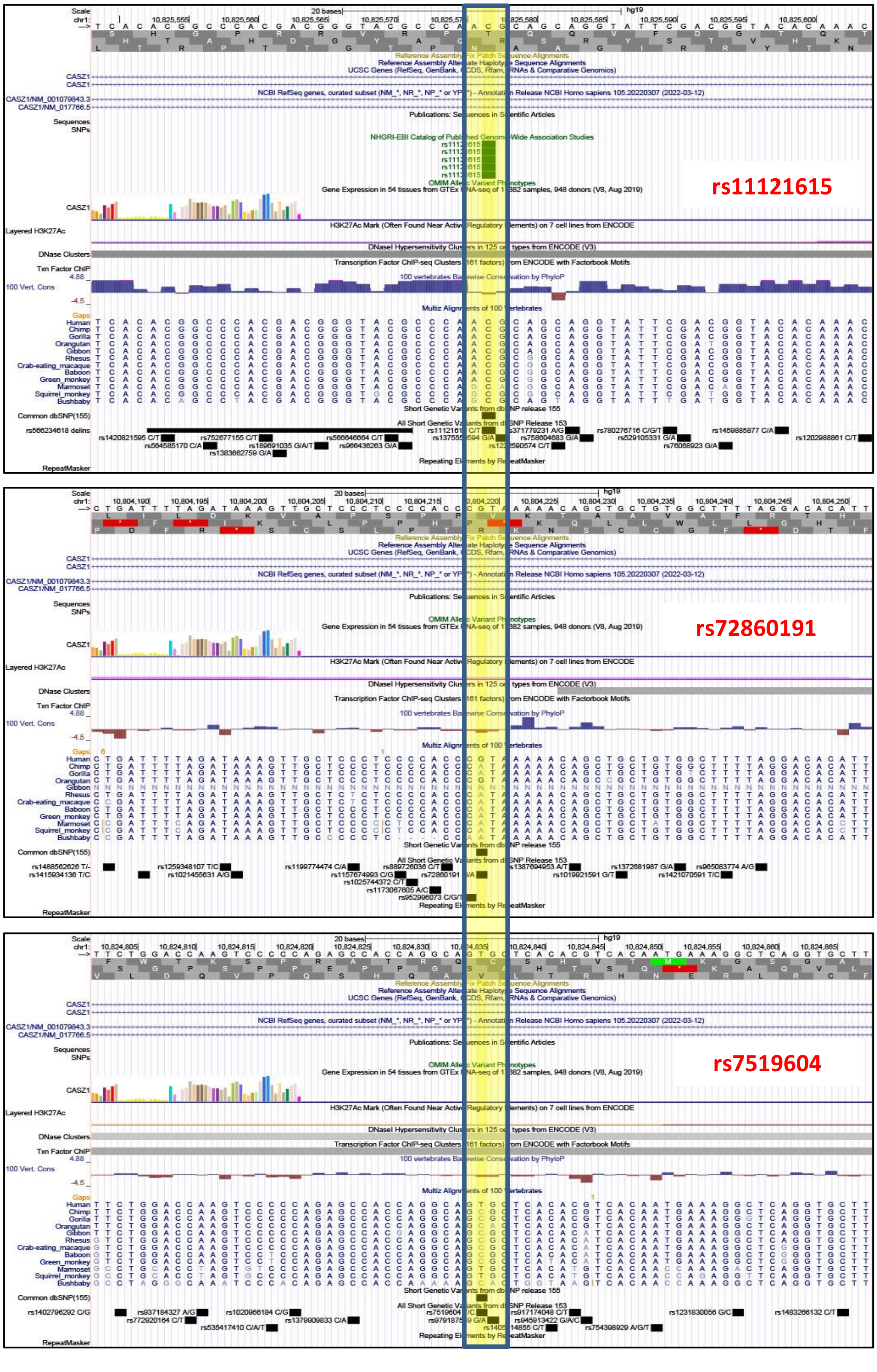

Supplementary figure 2: UCSC Genome Browser screenshots depicting the conservation at the significantly associated variation of *CASZ1* gene across Primates. The variations observed to be highly conserved in all the Primates and are highlighted in yellow color.

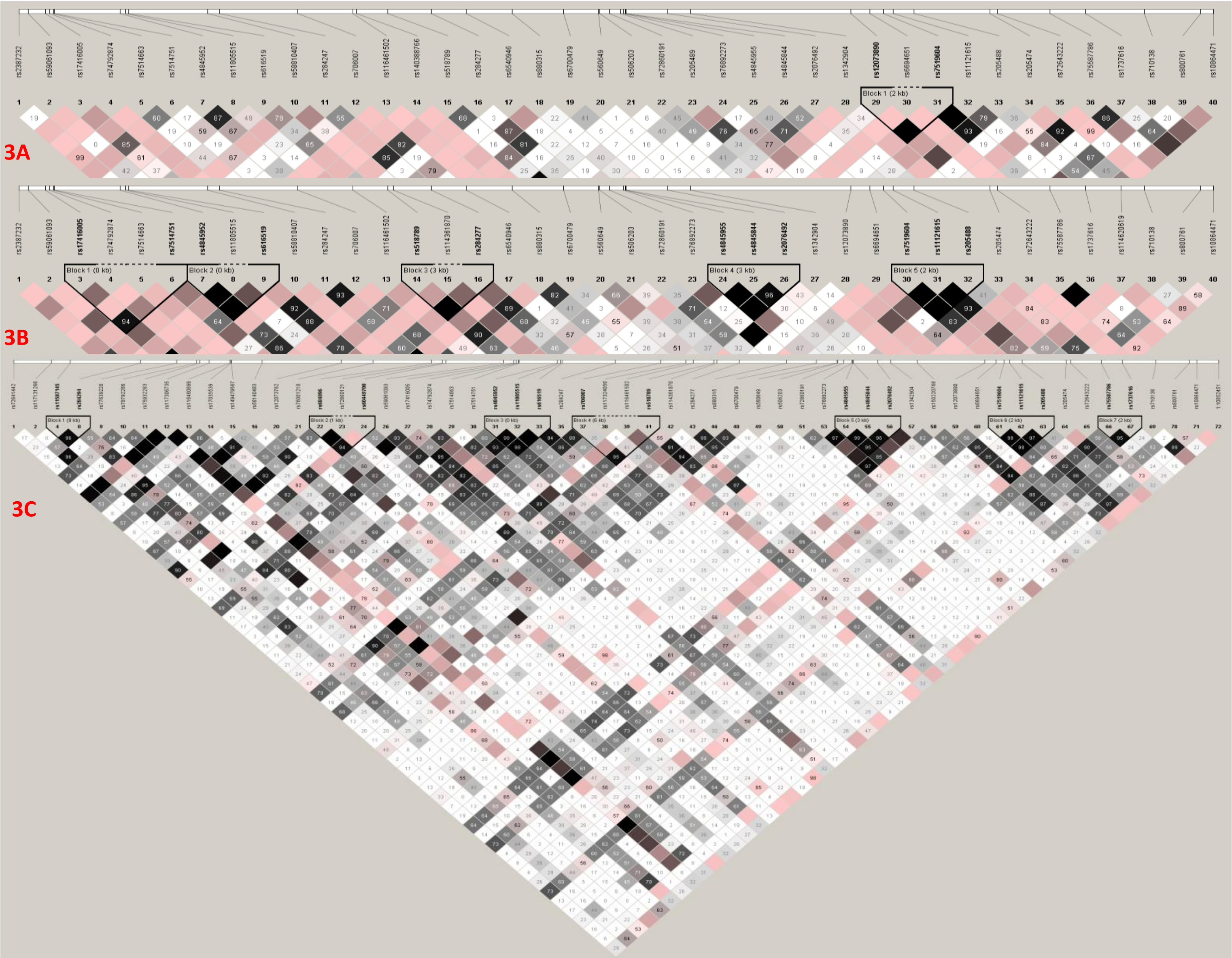

Supplementary figure (3A) represents the haplotype structure of *CASZ1* gene on the chr 1:10697392-10852451 in Africans; Figure (3B) represents the haplotype structure of *CASZ1* gene on the chr 1:10697392-10852451 in Europeans and Figure (3C) represents the haplotype structure of *CASZ1* gene on the chr 1:10697392-10852451 derived from present study in South Asian Indians. The data of the respective populations was derived from Ensembl.org using VCF to PED converter tool. The observed haplotypic structures of Africans and Europeans are different from South Asians; signifying an outcome of founder effect due to ancient admixture.
